# Strategies targeting endoplasmic reticulum stress to improve Parkinson’s disease

**DOI:** 10.3389/fphar.2023.1288894

**Published:** 2023-11-10

**Authors:** Danni Wang, Shuhui Qu, Zaijun Zhang, Liang Tan, Xiuping Chen, Hai-Jing Zhong, Cheong-Meng Chong

**Affiliations:** ^1^ State Key Laboratory of Quality Research in Chinese Medicine, Institute of Chinese Medical Sciences, University of Macau, Macao, China; ^2^ International Cooperative Laboratory of Traditional Chinese Medicine Modernization and Innovative Drug Development of Chinese Ministry of Education (MOE), College of Pharmacy, Jinan University, Guangzhou, China; ^3^ Department of Neurosurgery, Southwest Hospital, The Third Military Medical University (Army Military Medical University), Chongqing, China

**Keywords:** Parkinson’s disease, dopaminergic neurons, ER stress, unfolded protein response, protein homeostasis

## Abstract

Parkinson’s disease (PD) is a common neurodegenerative disorder with motor symptoms, which is caused by the progressive death of dopaminergic (DA) neurons in the substantia nigra pars compacta (SNpc). Accumulating evidence shows that endoplasmic reticulum (ER) stress occurring in the SNpc DA neurons is an early event in the development of PD. ER stress triggers the activation of unfolded protein response (UPR) to reduce stress and restore ER function. However, excessive and continuous ER stress and UPR exacerbate the risk of DA neuron death through crosstalk with other PD events. Thus, ER stress is considered a promising therapeutic target for the treatment of PD. Various strategies targeting ER stress through the modulation of UPR signaling, the increase of ER’s protein folding ability, and the enhancement of protein degradation are developed to alleviate neuronal death in PD models. In this review, we summarize the pathological role of ER stress in PD and update the strategies targeting ER stress to improve ER protein homeostasis and PD-related events.

## 1 Introduction

Parkinson’s disease (PD) is one of the most common neurodegenerative disorders, causing well-characterized symptoms such as bradykinesia, rigidity, resting tremors, and abnormal posture (Michel et al., 2016). It is caused by the progressive degeneration and death of dopaminergic (DA) neurons in the substantia nigra pars compacta (SNpc) in the midbrain (Jankovic and Tan, 2020). In addition to motor defects, PD patients have non-motor symptoms such as dyskinesia, cognitive disorder, psychosis, gastrointestinal disorder, etc. PD affects approximately 1% older population all over the world (Yu et al., 2022). The estimated number of populations diagnosed with PD would be over 12 million by 2040 (Dorsey and Bloem, 2018). At present, clinical management of motor symptoms in PD can be mainly divided into drug therapy and surgical treatment (Bloem et al., 2021; Alarcon et al., 2023). Pharmacological strategies can increase dopamine concentrations or stimulate dopamine receptors to improve motor symptoms and quality of life in PD patients (Bloem et al., 2021). Deep brain stimulation is the most effective surgery for alleviating PD motor symptoms through the electrical effects of stimulation (Groiss et al., 2009). It is an option for those patients no longer controlled well by dopamine-related drugs. However, current PD treatment provides symptomatic relief for the motor problems but does not prevent or slow the process of PD (Mondal et al., 2023).

Up to now, the exact etiology and mechanism of PD remain arguments. The incidence and symptoms of PD are highly associated with age; thus, aging is considered a main factor for PD development. There is a piece of evidence to support that genetic and environmental factors contribute to PD development. Most cases belong to sporadic PD; less than 10% of PD patients have an inherited history. These familial PD cases result from the mutations in a group of genes such as α-synuclein (*SNCA*), PTEN-induced putative kinase 1 (*PINK1*), parkin (*PARK2*), Vacuolar protein sorting ortholog 35 (*VPS35*), DJ-1 (PARK7), glucocerebrosidase (*GBA*), phospholipase A2 group 6 (*PLA2G6*), Leucine-rich repeat kinase 2 (*LRRK2*), lipoprotein receptor-related protein 10 (*LRP10*), ubiquinol cytochrome c reductase (*UQCRC1*), etc (Korecka et al., 2019; Jia et al., 2022; Li et al., 2022). In addition to aging and genetic factors, accumulated evidence shows that PD may result from environmental risk factors such as pesticides and other toxin exposure, head injury, diet, smoking, stress, etc (Steece-Collier et al., 2002; Marras et al., 2019). Moreover, type 2 diabetes mellitus (T2DM) patients with age over 65 years were more likely to exhibit an increased risk of PD (Yang et al., 2017). However, how these risk factors lead to PD is poorly understood.

The endoplasmic reticulum (ER) is in charge of protein biosynthesis, folding, and assembly. Once the ER is damaged or its protein folding capacity is overloaded, it will accumulate unfolded/misfolded proteins in the ER, which is called ER stress. To improve ER protein homeostasis and restore the function of ER, cells result in unfolded protein response (UPR) to activate the signaling network (Hetz, 2012). However, continuous ER stress and UPR activation are known to induce cell death and are observed in neurodegenerative diseases (Hoozemans et al., 2007; Wang et al., 2023). SNpc DA neurons often face stress from high energy requirements, high levels of reactive dopamine, and calcium ions (Ca^2+^) handling, revealing that faster protein turnover in these neurons is required to cope with these problems. One evident hallmark of PD is Lewy bodies mainly consisting of misfolded α-synuclein. In PD patients, this abnormal protein aggregate accompanying dominant ER stress could be observed in DA neurons of the SNpc (Hoozemans et al., 2007), suggesting that ER stress may exacerbate the risk of DA neuron death through crosstalk with other PD events. Notably, several studies have demonstrated that some small molecule compounds can reduce ER stress to protect DA neurons in the PD model; thus, ER stress has been considered a promising therapeutic target for PD. In this review, we briefly summarize the recent studies regarding the role of ER stress in PD development and explore the potential of strategies targeting ER stress for PD.

## 2 ER stress triggers UPR

**FIGURE 1:**
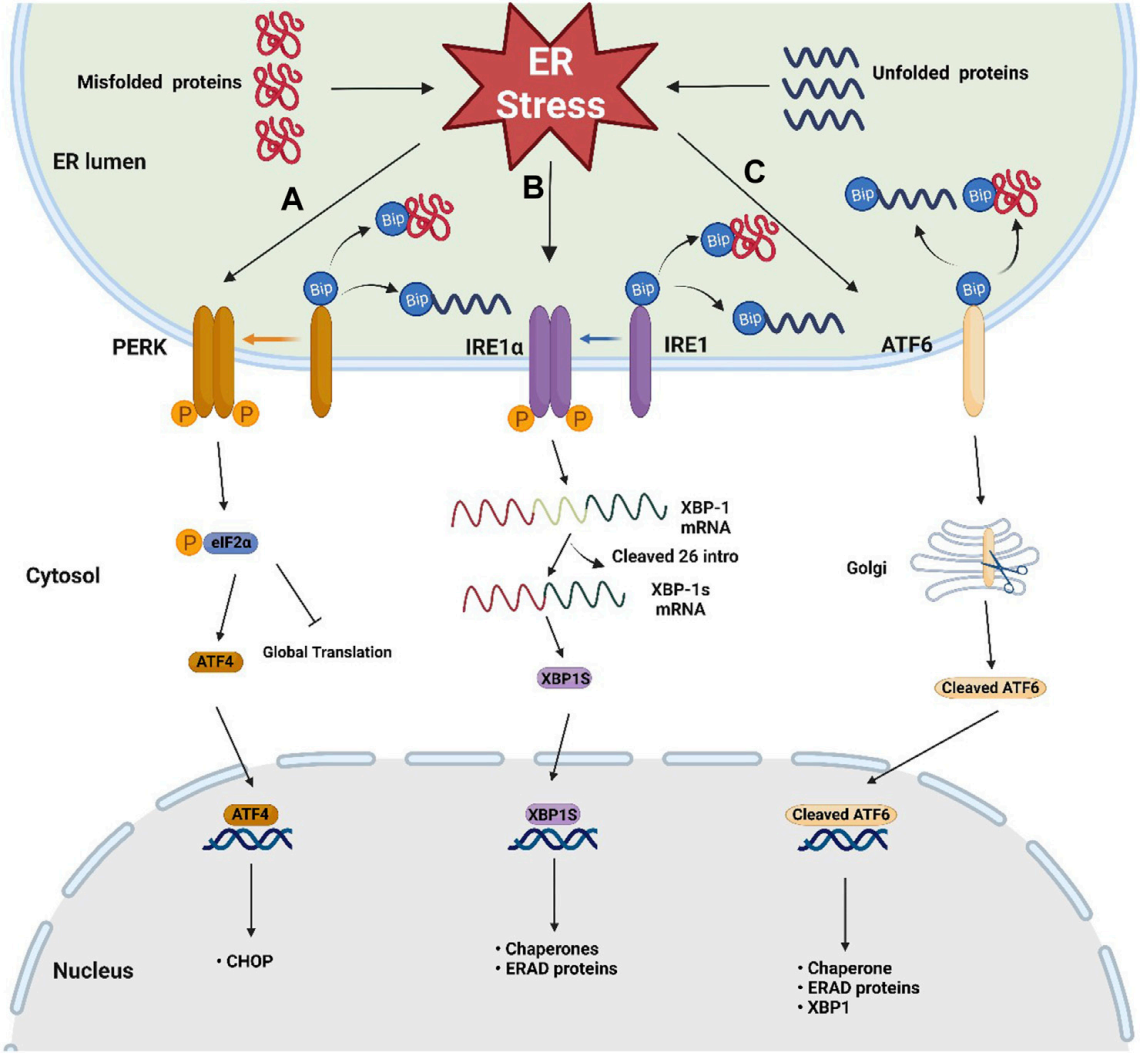
Schematic representation of the three sensors of UPR. Three key UPR pathways after the dissociation of BiP from three sensors when ER stress occurs: **(A)** PERK/eIF2α/ATF4 pathway: trans autophosphorylation of the PERK leads to phosphorylation of eIF2α, then attenuating global protein translation and increases ATF4 translation, **(B)** IRE1/XBP-1 pathway: the phosphorylated dimer of the IRE1α with RNAase activity cleaves out 26 intronic nucleotides of the XBP-1 mRNA, resulting in the generation of the XBP1s, which is responsible for inducing gene expression involved in protein folding and ERAD, **(C)** ATF6 pathway: ATF6 migrates to the Golgi apparatus, where it becomes active by protease cleavage. Active ATF6 translocates to the nucleus and induces the expression of genes involved in chaperone, XBP-1, and ERAD.

The ER is the largest and most complicated organelle in eukaryotic cells, consisting of an interconnected membrane network involved in a series of cellular functions, including biosynthesis, folding, post-translational modification, and assembly of protein as well as intracellular Ca^2+^ homeostasis and lipid metabolism (Schwarz and Blower, 2016; Zhao et al., 2023). ER stress occurs when the ER is overwhelmed by the accumulation of unfolded or misfolded proteins (Jia et al., 2022) and then activates the UPR, a signaling cascade for restoring protein homeostasis (Hetz et al., 2020). The UPR is a complex, adaptive response that triggers the activation of three key pathways: the protein kinase RNA-like endoplasmic reticulum kinase (PERK)/eukaryotic initiation factor 2 alpha (eIF2α)/activating transcription factor 4 (ATF4) pathway, the inositol-requiring enzyme 1 (IRE1)/X-box binding protein-1 (XBP1) pathway, and the activating transcription factor 6 (ATF6) pathway (Frakes and Dillin, 2017) (Figure 1). In the ER lumen, PERK, IRE1α, and ATF6 are important sensors in cellular defense against ER stress (Ghemrawi and Khair, 2020). In general, these UPR senor proteins located in the ER membrane are bound to the ER-resident chaperone immunoglobulin-binding protein (BiP/GRP78) (Ghemrawi and Khair, 2020). BiP is the Hsp70-type chaperone for helping protein folding and plays a central role in regulating these UPR sensors’ state (Pobre et al., 2019).

When ER stress occurs, the accumulation of misfolded proteins causes the dissociation of BiP from these sensors, leading to UPR activation (Ghemrawi and Khair, 2020). After dissociation from BiP, the phosphorylated dimer of the cytoplasmic domain of IRE1α has RNAase activity, which can cleave out 26 intronic nucleotides of the mRNA encoding XBP-1, resulting in the generation of the active transcription factor XBP1s, which is responsible for inducing gene expression involved in protein folding and ER-associated protein degradation (ERAD) (Park et al., 2021; Siwecka et al., 2021). After BiP dissociation, trans autophosphorylation of the PERK C-terminal cytoplasmic kinase domain leads to phosphorylation of eIF2α. Phosphorylated eIF2α attenuates global protein translation and increases ATF4 translation (Ghemrawi and Khair, 2020). After release from BiP, ATF6 migrates to the Golgi apparatus, where it becomes active by protease cleavage. Active ATF6 translocates to the nucleus and induces the expression of genes involved in chaperone, XBP-1, and ERAD (Wang et al., 2000). In addition to the crosstalk between the ATF6 pathway and the IRE1-XBP1 pathway, studies have shown that there is also crosstalk between all three pathways. In cardiomyocytes and Hela cells, coxsackievirus B3 can activate the ATF6a/IRE1-XBP1 pathway, thereby downregulating p58 IPK expression. Downregulated p58 IPK will further activate the PERK pathway to bridge the three pathways (Zhang et al., 2010). Crosstalk between different UPR branches is important for understanding the overall mechanism of ER stress and UPR. However, there are not many studies focusing on this aspect, especially in neurons, and more exploration is needed. Notably, when the UPR fails to address the ER stress, it promotes more ATF4 translation through phosphorylated eIF2α, thereby enhancing the upregulation of C/EBP homologous protein (CHOP), initiating apoptotic pathway (Ghemrawi and Khair, 2020).

## 3 ER stress contributes to PD

### 3.1 PD patients

Clinical reports indicate that ER stress is highly correlated with PD pathogenesis. In the SNpc of PD patients, the markers of UPR activation (p-PERK, p-eIF2α, and p-IRE1α) were observed in DA neurons (Hoozemans et al., 2007; Heman-Ackah et al., 2017b). It should be noted that the p-PERK and p-IRE1α had colocalization with increased α-synuclein in PD DA neurons (Hoozemans et al., 2007; Heman-Ackah et al., 2017b), revealing that UPR activation is highly associated with the accumulation of misfolded α-synuclein in PD. Moreover, in the postmortem brain tissue of PD patients, BiP levels significantly reduced in the temporal cortex and cingulate gyrus, but not in the caudate, prefrontal, or parietal cortex regions (Baek et al., 2019), demonstrating that the ER’s protein folding function is decreased in certain brain regions of PD patients. However, in the SNpc of PD patients, ER stress response proteins including BiP, homocysteine-induced endoplasmic reticulum protein (Herp), and protein disulfide isomerase (PDI) were increased and co-localized with α-synuclein (Conn et al., 2004; Slodzinski et al., 2009; Selvaraj et al., 2012), revealing that chronic ER stress occurs in patients’ SNpc.

### 3.2 Neurotoxin-induced PD models

The parkinsonism-inducing neurotoxins such as 6-hydroxydopamine (6-OHDA), 1-methyl-4-phenyl-pyridinium (MPP^+^)/1-methyl-4-phenyl-1,2,3,6-tetrahydropyridine (MPTP), and rotenone are commonly used for the establishment of various PD models. The main neurotoxicity of 6-OHDA results from its oxidant activity; MPP^+^ and rotenone are mitochondria-targeting PD neurotoxins. A piece of evidence suggests that these neurotoxins can cause ER stress. In SNpc of rabbits, administration of MPP^+^ could induce ER stress through activation of the ATF-6 pathway (Ghribi et al., 2003). In 6-OHDA-induced PD rats, increased levels of BiP and CHOP were observed (Ning et al., 2019). Similarly, 6-OHDA treatment enhanced ER stress-related proteins in rat neuron culture (Cai et al., 2016). However, in some cell line-based studies, 6-OHDA was found to induce the activation of three UPR branches, whereas MPP^+^ or rotenone only triggered IRE1α and PERK/ATF4 activation (Ryu et al., 2002; Holtz and O'Malley, 2003). On the other hand, in rat SN, rotenone caused a significant decrease in the expression of parkin, PARK 7 (DJ1), and C terminus Hsp70 interacting protein (CHIP), accompanying the increase in ubiquitin (Sonia Angeline et al., 2012), revealing that ubiquitin-proteasome system (UPS) exhibits the impairment in this PD-liked model. UPS dysfunction leads to protein accumulation, and may trigger ER stress during PD development. The structure of herbicide paraquat is quite similar to MPP^+^ and has been found to inhibit UPS and thereby lead to ER stress-induced apoptosis in rat mesencephalic DA N27 cells (Chinta et al., 2008). Paraquat and rotenone appear to have similar mechanisms for ER stress. These PD-associated toxins cause ER stress and UPR; however, the mechanisms by which they activate the UPR pathway vary.

### 3.3 PD-related gene mutations

Studies of familial PD-related genes have shown that ER stress-related mechanisms and cellular pathways are essential for PD development. In PC12 cell lines, the α-synuclein A53T mutant overexpression decreased proteasome activity as well as increased the reactive oxygen species (ROS) levels and apoptosis (Smith et al., 2005). ER stress and caspase-12 activation also occurred in PC12 cells with α-synuclein A53T mutant, whereas an ER stress inhibitor knocked down caspase-12 levels or partially increased cell survival (Smith et al., 2005), revealing that ER stress involved in the α-synuclein A53T mutant-induced cell death. In the rat, excess exogenous α-synuclein triggered the activation of UPR pathways and apoptosis in SNpc DA neurons, whereas overexpression of BiP chaperone could reduce excess α-synuclein-induced neurotoxicity by reducing UPR activation (Gorbatyuk et al., 2012). Speal receptors (SR) can be ubiquitinated by Parkin’s ubiquitin-protein ligase activity (Imai et al., 2001). Highly insoluble SR aggregation was observed in the brain with juvenile PD (Imai et al., 2001). Lmai et al. demonstrated that highly insoluble SR aggregation could lead to UPR activation, which may contribute to the loss of DA neuronal (Imai et al., 2001). Astrocytes play a role in supporting neuronal function and are highly implicated in the pathogenesis of PD (Karpinar et al., 2009; Lim et al., 2009; Halliday and Stevens, 2011; Meares et al., 2014). Astrocytes that take up pathological α-synuclein released from axon terminals produce chemokines and pro-inflammatory cytokines. These cytokines could trigger microglial activation and ultimately cause neuronal degeneration (Lee et al., 2010). In addition, in mice astrocytes carrying LRRK2-G2019S mutant, α-synuclein mutant led to Ca^2+^ depletion in the ER lumen by interacting with SERCA, which caused ER stress and subsequent cell loss (Lee et al., 2019).

Since human PD brain samples are limited, PD patient induced pluripotent stem cells (iPSCs) give an alternative human model to study PD mechanisms, particularly in familial PD gene mutations. In the neurons derived from PD patient iPSCs carrying α-synuclein A53T mutation, Chung et al. found that the hallmark of ER stress, PDI increased, but the marker of ER stress-induced cell death, CHOP, did not have the change (Chung et al., 2013), revealing that these mutant neurons are still at an early pathologic stage. In the PD patient iPSC-derived DA neurons with GBA N370S mutation, Fernandes et al. found that misprocessing of mutant GBA protein in the ER was positively correlated with ER stress (Fernandes et al., 2016). In DA neurons from the patient iPSCs carrying homozygous PLA2G6 D331Y mutation, Ke et al. (2020) found that abnormal ER-mediated Ca^2+^ homeostasis and ER stress increase with culture time, accompanied by neuronal death. In addition, they found that ER stress was associated with mitochondrial dysfunction and neuronal death. These studies demonstrated that ER stress is commonly induced by these familiar PD gene mutants and may result from the dysfunction of protein hemostasis. Notably, a recent study reveals that aggregated α-synuclein caused patient DA neurons unresponsive to ER stress (Stojkovska et al., 2022). They found that in DA neurons derived from PD patient iPSCs carrying 3X *SNCA*, increased α-synuclein caused ER fragmentation and protein folding ability decrease, leading to the accumulation of insoluble immature β-glucocerebrosidase (GCase) in ER (Stojkovska et al., 2022). However, collapsed protein homeostasis did not induce UPR activation, hinting that increased α-synuclein led to the dysfunction of the ER stress sensor through an unknown mechanism.

### 3.4 Interaction between ER stress and other PD pathological events

Pathological aggregation of α-synuclein, neuroinflammation, oxidative stress, and mitochondrial dysfunction are common features of PD. They have crosstalk with ER stress, which jointly exacerbates the process of PD. As mentioned above, UPR activation is closely related to α-synuclein aggregation. Pathological α-synuclein can interact with BIP, which separates BIP from the three UPR effector proteins, thereby activating the UPR pathway (Bellucci et al., 2011). In addition, the accumulation of pathological α-synuclein can also induce ER stress by impairing autophagy and the UPS system, thereby activating the UPR (Kim et al., 2022). Correspondingly, ER stress can also upregulate the release of α-synuclein from neurons and the transmission between neurons to cause deposition in multiple brain regions, leading to neuroinflammation, thereby aggravating the PD process (Jang et al., 2010).

Active microglia and astrocytes are the major sources of neuroinflammation, and their persistent pathological neuroinflammation is known to promote neuronal death (Glass et al., 2010; Prinz and Priller, 2014; Sofroniew, 2015). Compared with neurons, microglia and astrocytes are highly resistant to ER stress-induced cell death (Meares et al., 2014). The relationship between ER stress and neuroinflammation is mainly linked by the PERK pathway. When ER stress occurs, PERK-mediated phosphorylation of eIF2α leads to the nuclear translocation of B cell nuclear factor kappa light chain enhancer (NF-κB) and thereby enhances the expression of its downstream inflammatory genes (Deng et al., 2004; Wu et al., 2018b). In addition, the activation of the PERK pathway in astrocytes also induces the JAK/STAT3 pathway to enhance the expression of chemokines and interleukin 6 (IL-6). Active astrocytes further trigger microglial activation via PERK-mediated paracrine signaling (Meares et al., 2014).

Electron microscopy has demonstrated that parts of the ER are tightly attached to mitochondria, forming a highly dynamic contact site named the mitochondria-associated membrane (MAM). MAM is widely present in brain neurons, where a variety of proteins such as calcium channels, vesicle-associated protein, sigma 1 receptor, apolipoprotein B, and other secreted proteins play an important role in cell survival (Sunanda et al., 2021; Markovinovic et al., 2022). The UPR-sensing protein PERK has also been found to aid apoptosis by transferring ROS signals from the ER to mitochondria through MAM (Sassano et al., 2021). One of the main functions of MAM is to transmit Ca^2+^ (Gomez-Suaga et al., 2018). In a healthy state, the process of Ca^2+^ flowing from ER to mitochondria is tightly regulated (Chou et al., 2023). The Ca^2+^ homeostasis is important for the functions of DA neurons (Salthun-Lassalle et al., 2004; Chan et al., 2007). Neurotoxin-induced ER stress and pathological depletion of ER Ca^2+^ storage lead to uncontrolled Ca^2+^ transport (Kim et al., 2022). Ca^2+^ above or below the threshold is taken up by mitochondria, leading to mitochondrial dysfunction (Cali et al., 2011; Bravo et al., 2013). Furthermore, mitochondrial dysfunction generates abnormal amounts of ROS, which in turn results in harmful oxidative damage to neurons (Karvandi et al., 2023).

Recent reviews provide a good summary of the basis on which PD not only causes dysproteostasis but also lipid dyshomeostasis (Yang et al., 2022; Flores-Leon and Outeiro, 2023). Many studies have shown that *GBA1* mutations are associated with an increased risk of PD, resulting in GCase misfolding and retention in the ER. These *GBA1* mutations also lead to loss of GCase activity and accumulation of its lipid substrates in the lysosome, consequently impairing the lysosomal function (Leyns et al., 2023), revealing that there is a connection between ER and lipid dyshomeostasis in PD. In addition to ER protein homeostasis, ER also plays the important role in lipid metabolism. Thus, alterations in lipid homeostasis also can affect ER function and trigger ER stress. This view is demonstrated by a recent study. *let-767* is a putative hydroxysteroid dehydrogenase in *Caenorhabditis elegans* for maintaining ER protein and lipid homeostasis. Loss of *let-767* was reported to lead to the accumulation of fatty acid metabolites, which in turn disrupted lipid homeostasis and ER function, then inducing UPR (Garcia et al., 2023). In addition, excessive accumulation of triacylglycerol in macrophages activated the UPR, leading to activation of the BIP/PERK/ATF4/Chop pathway and translocation of ATF6 into the nucleus (Aflaki et al., 2012).

Glycosylation is an abundant post-translational modification (PTM), particularly on membrane-bound and lysosomal proteins, and its dysregulation has also been found in PD brains (Schneider and Singh, 2022). In addition to protein synthesis, the ER also regulates glycosylation and functionalization of proteins. Abnormal glycosylation is known to lead to abnormal protein deposition, causing ER overload and triggering ER stress (Schneider and Singh, 2022).

The above articles objectively study the crosstalk between ER stress and other important pathological processes of PD, providing a broader perspective for discovering PD intervention strategies. However, more research is still needed to fully elucidate the fundamental mechanism of this interaction to deal with PD. Some studies have limitations of the research model. For example, the link between lipid homeostasis and ER proteostasis is currently more established in non-neurons. Moreover, there are still few studies on the connection between glycosylation dysregulation and ER stress; thus, more evidence is needed to elucidate their relevance.

## 4 Strategies to improve PD by targeting ER stress

Given ER stress plays a critical role in PD development, it has been considered a potential therapeutic target for PD. Protein homeostasis dysfunction is the most critical cause and the most direct phenomenon of ER stress. Therefore, several strategies targeting ER stress mainly focus on the modulation of UPR signaling, the increase of ER’s protein folding ability, and the enhancement of protein degradation (Table 1).

**TABLE 1:** Three strategies to improve PD by targeting ER stress.

Type	ER stress regulator	Target/proved mechanism	Effects	Clinical trials
Modulation of UPR signaling	GSK2606414	PERK inhibitor	Protecting DA neurons, increasing dopamine levels, and improving the motor deficits	-
	LDN-87357	PERK inhibitor	Reducing ER stress markers, increasing cell viability, and reducing apoptosis	-
	Salubrinal	Dephosphorylation inhibitor of elF2α	Protecting Tm and BFA-treated PC12 cell line from apoptosis, and protecting DA neurons	-
	MANF	IRE1α	Protecting neurons	-
	CDNF	IRElα and PERK pathways regulator	Protecting neurons	Increasing availability of DAT in the putamen with slower disease progression in the first phase I-II clinical tests
	AAV-loaded XBP1s	XBP1s	Suppressing 6-OHDA- and MPTP-induced degeneration of DA neurons in PD mice	-
	Liraglutide	ATF6	Reducing ER stress and providing neuroprotective effects	Improving non-motor function and activities of daily living in PD patients in a phase II trial
	AAV-loaded UPRplus	ATF6f/XBP1s fusion protein	Reducing abnormal aggregation of mutant α-synuclein	-
Increase of ER's protein folding ability	Azoramide	BiP	Reducing ER stress, mitochondria dysfunction, oxidative stress, and cell death in PD patient iPSC-derived DA neurons	
	Baicalein	BiP	Reducing 6-OHDA, MPP+, MPTP, Tg, BFA-induced neurotoxicity	-
	GIF-0854-r and GIF-0856-r	Chemical chaperone	Reducing protein aggregation and ultimately attenuated cell death	-
	4-PBA	Chemical chaperone	Reducing rotenone-induced oxidative stress, a-synuclein increase, mitochondrial dysfunction, and DA neuronal death in rotenone-induced PD rat model	-
Enhancement of protein degradation	A-443654	Autophagy	Reducing α-synuclein in DA neurons	-
	Empagliflozin	Autophagy	Reducing accumulation of α-synuclein	-
	Naringenin	UPS	Decreasing of ubiquitination level, thereby increasing the degradation of misfolded proteins	-
	Puerarin	UPS	Restoring the normal function of UPS	-

### 4.1 Modulation of UPR signaling

Continuous activation of UPR branch signaling has been observed in the PD patient’s DA neurons. It is highly associated with DA neuronal death; thus, the modulation of the UPR signaling pathways may be beneficial in suppressing DA neuronal death and PD-related phenomena.

#### 4.1.1 PERK/eIF2α/ATF4 pathway

GSK2606414 was found to be a selective PERK inhibitor. It was reported to be able to crossover BBB and protect DA neurons against 6-OHDA toxicity in mice (Mercado et al., 2018). In addition, GSK2606414 could increase dopamine levels, restore the synaptic proteins, and improve motor deficits in mice (Mercado et al., 2018). Small-molecule PERK inhibitor LDN-87357 successfully reduced mRNA levels of ER stress markers and caspase3-associated apoptosis in SH-SY5Y cell PD model (Lusa et al., 2023). On the other hand, to regulate the UPR branch, Boyce et al. discovered a “different way” of the low-toxicity compound Salubrinal (Boyce et al., 2005). It could inhibit the dephosphorylation of eIF2α, and increase eIF2α phosphorylation to upregulate the expression of downstream protein CHOP. The pharmacological effect of dephosphorylation was considered rare and difficult at that time. In their study, Salubrinal protected rat pheochromocytoma cell line PC12 from tunicamycin (Tm)- and brefeldin A (BFA)-induced cell damage without causing ER stress. Furthermore, this dephosphorylation inhibition was independent of eIF2α upstream kinases. Gupta et al. used a rotenone-induced PD rat model and verified that the prosurvival effect of Salubrinal was via regulating ER stress and UPR, showing that dephosphorylation of eIF2α by Salubrinal confers the protective effect against rotenone-induced neuronal death (Gupta et al., 2021).

#### 4.1.2 IRE1/XBP1 pathway

Mesencephalic astrocyte-derived neurotrophic factor (MANF) and cerebral dopamine neurotrophic factor (CDNF) are two types of neurotrophic factors distributed in the ER lumen (Lindholm and Saarma, 2022). Some studies showed that they express the activity to protect DA neurons via regulating UPR signaling. unlike other known neurotrophic factors, CDNF and MANF are not secreted proteins and have highly similar protein structures. Recently, Kovaleva et al. used Tm-induced mice primary SCG neurons and a 6-OHDA-induced PD rat model to demonstrate that MANF confers protective effects in neurons (Kovaleva et al., 2023). MANF could compete with BIP for binding to IRE1α with high affinity, regulating its oligomerization, phosphorylation, and downstream signal transduction (Kovaleva et al., 2023). CDNF was shown to be a UPR protein in mice models of Tm-induced ER stress and primary neuronal models (Eesmaa et al., 2022). Its expression is regulated by ER stress *in vivo* and can regulate IRE1α and PERK pathways to protect against apoptosis in neurons (Eesmaa et al., 2022). In addition, researchers also found that CDNF may interact with the BiP-GRP170 complex and that CDNF was a cofactor rather than a substrate of BiP (Eesmaa et al., 2022). Following preclinical studies and toxicity studies in various small and large animal models of PD, the first phase I-II clinical tests of CDNF in PD patients were performed (Huttunen and Saarma, 2019). In the test, intraputamenal human CDNF infusions showed good safety and tolerability, meeting the primary endpoint. Furthermore, increased availability of dopamine transporter (DAT) in the putamen was also observed in the CDNF-receiving group, with slower disease progression.

Adeno-associated virus (AAV)--based gene therapy has the advantages of safety and stability. Previously, the researchers showed that exogenous brain injection of AAV-loaded XBP1s significantly suppressed 6-OHDA- and MPTP-induced loss of DA neurons in mice via up-regulating its downstream UPR genes (Sado et al., 2009; Valdes et al., 2014).

#### 4.1.3 ATF6 pathway

Liraglutide is a glucagon-like peptide 1 receptor agonist for the treatment of T2DM. Liraglutide reduced palmitate-induced ATF6 nuclear translocation and prevented the changes in ER proteostasis and ER morphology in Neuro2A cells (Griffin et al., 2022). Liraglutidea exhibited the potency to reduce ER stress (Panagaki et al., 2017; Breton-Romero et al., 2018). It can bypass the -blood-brain barrier (BBB) and provide neuroprotective effects in various PD models (Athauda and Foltynie, 2016). A recent clinical study showed that the non-motor symptoms and activities of daily living in PD patients can be improved by liraglutide treatment (Malatt et al., 2022).

Later, Vidal et al. developed an ATF6f/XBP1s fusion protein called UPRplus. UPRplus could regulate the expression of downstream genes via enforced dimerization of the XBP1s and ATF6f domains. It found that AAV-loaded UPRplus was more potent in decreasing abnormal aggregation of mutant α-synuclein than either ATF6 or XBP1 alone (Vidal et al., 2021). Given the functions of XBP1s and ATF6f in upregulating the expression of chaperone and ERAD-related genes, AAV-loaded UPRplus can promote ER’s protein folding and protein degradation to maintain protein homeostasis.

In the study of UPR branches alleviating ER stress, all three branches have received relatively comprehensive attention from scientists, and many small molecule compounds that can regulate these branches have been developed. In addition, many screening methods targeting UPR branches have also emerged. However, there are relatively few studies on clear targets, and researchers often ignore the connections between different branches in the action of compounds, and the selectivity of many compounds is not studied in depth enough.

### 4.2 Increase of ER’s protein folding ability

α-synuclein accumulation leads to the decrease of ER chaperone level in DA neurons derived from PD patient iPSCs carrying 3X *SNCA* gene (Stojkovska et al., 2022), hinting that ER’s protein folding ability declines during PD development. Thus, increasing ER chaperone may avoid the protein accumulation in ER, and thereby reduce the development of various PD phenomena.

Azoramide is a small molecule compound that can improve protein folding and increase BiP chaperone expression against ER stress (Fu et al., 2015). Our team found that Azoramide could not only reduce the activation of UPR branches but also effectively alleviate the loss of familial PD patient DA neurons (Ke et al., 2020). Through mechanism exploration, it was found that Azoramide could reduce the increase of ROS, and mitochondrial fragmentation, as well as improve the decline of mitochondrial membrane potential. In addition, Azoramide could resume Ca^2+^ homeostasis in patient DA neurons by mediating store-operated Ca^2+^ entry (SOCE) and ER function.

Chemical chaperones are a class of small molecules that act to increase protein re-folding and suppress protein aggregates. Hasegawa et al. identified two novel oxindole compounds, GIF-0854-r and GIF-0856-r, with chaperone activity which inhibited BSA aggregation and reduce protein aggregation caused by Tm-induced ER stress in hippocampal HT22 neurons (Hasegawa et al., 2022). Furthermore, these two compounds inhibited downstream molecules of the UPR pathway and ultimately attenuated cell death (Hasegawa et al., 2022). There are two chemical chaperones with therapeutic promise in PD. 4-phenyl butyric acid (4-PBA) is a well-documented chemical chaperone that can prevent the misfolding and mislocalization of proteins. Recent studies show that 4-PBA exhibited significant neuroprotective effects in the rotenone-induced PD rat model (Tiwari et al., 2022). In both SNpc and striatal regions of the rat PD model, they found that 4-PBA administration reduced rotenone-induced oxidative stress, α-synuclein increase, mitochondrial dysfunction, and DA neuronal death.

Different neurotoxins have different effects on the chaperone BIP due to different toxicity mechanisms. MPP^+^ has been shown to reduce the expression of BIP in SH-SY5Y cells (Ai et al., 2023), while 6-OHDA has been shown to increase the expression of BIP in neurons (Holtz and O'Malley, 2003). Therefore, more chaperones should be included in studies to discover more effective compounds, such as chaperone PDI and Sigma1R (Perri et al., 2015; Voronin et al., 2023). In addition, the improvement of protein folding by adding chaperones should be verified through more aspects such as electron microscopy observation or structural analysis after protein purification, to increase the convincingness.

### 4.3 Enhancement of protein degradation

Autophagy is a self-eating process that can degrade the misfolded/unfolded proteins from the ER and damaged ER (Chipurupalli et al., 2021). In addition, autophagy can remove accumulated α-synuclein, which may benefit the recovery of ER function. Some small molecules have been reported to regulate autophagy to reduce ER stress in PD models. A-443654, an inhibitor of the serine/threonine kinase Akt, could reduce α-synuclein in DA neurons differentiated from iPSCs with 3X *SNCA*, and restore the expression of autophagy-related genes (mTOR, p62, and LC3-II) and UPR-related proteins (BiP and CHOP) to non-stressed levels (Gandelman et al., 2021). Empagliflozin, a selective sodium-glucose cotransporter 2 inhibitor, could attenuate SNpc neuron death, neuroinflammation, and behavioral abnormalities in rotenone-induced PD rats. It was found that Empagliflozin enhanced autophagy and UPS by increasing beclin-1 protein, thereby reducing ER stress, and accumulation of α-synuclein in striatal of PD rats (Motawi et al., 2022). Mitophagy is also an important pathological organelle clearance process. Studies have shown that PINK1 can increase its expression by phosphorylating XBP1s *in vivo* and *in vitro*, thereby increasing mitophagy function and improving mitochondrial function (El Manaa et al., 2021).

ERAD involves recogniting abnormal proteins by ER chaperones that transfer damaged proteins from the ER to the cytoplasm, where they are modified with ubiquitin and delivered to the proteasome. In addition, like autophagy, UPS is involved in the degradation of accumulated α-synuclein as well. Some native compounds such as naringenin and puerarin have been found to regulate UPS well in PD models. In the rotenone-induced PD rat model, the administration of naringenin had promising effects on reducing cell death and motor deficits. Naringenin also could increase the level of the ubiquitin E3 ligase parkin and other related chaperones accompanying the decrease of ubiquitination level, thereby increasing the degradation of misfolded proteins (Sonia Angeline et al., 2013; Wu et al., 2018b). In the MPTP-induced PD mice model, naringenin was found to decrease α-synuclein levels and neuroinflammation (Mani et al., 2018), revealing that enhancing UPS may contribute to the protective effects of naringenin against these PD neurotoxins. Puerarin could reduce MPP^+^-induced morphological changes, cell death, and α-synuclein increase in SH-SY5Y cells (Cheng et al., 2009). In addition, Puerarin could upregulate the proteasome activity to remove the ubiquitin-conjugated proteins, thereby restoring the normal function of UPS (Cheng et al., 2009). Given the role of α-synuclein on ER stress, naringenin and puerarin may restore ER function by clearing α-synuclein through UPS.

## 5 Discussion

The above contents prove the rationality and applicability of ER stress as a potential therapeutic target for PD. However, there are some disadvantages of strategies targeting ER stress. For example, Salubrinal is an effective anti-ER stress compound through inhibiting dephosphorylation of elF2 α. However, the phosphorylation of eIF2α inhibits the overall synthesis of other proteins except for ATF4. The cells are in a state of being unable to synthesize necessary proteins for a long time, which obviously cannot meet the normal requirement for neuronal survival. This view is supported by the study from Halliday et al. They found that in the mouse prion model, the protective effect of Salubrinal was lost and even resulted in the death of neurons (Halliday and Mallucci, 2014). In addition, it is reported that sustained phosphorylation of eIF2α impairs memory function (Costa-Mattioli et al., 2007). Since PD is a chronic disease, it should be concerned the amount and timing of these UPR modulators.

Moreover, many fluorescent reporter systems targeting UPR are used for drug screening (Iwawaki et al., 2004; Furukawa and Xiong, 2005; Lajoie et al., 2014; Chaveroux et al., 2015; Fu et al., 2015; Heman-Ackah et al., 2017a; Wu et al., 2018a; Grandjean et al., 2020; Santiago-Lopez et al., 2022b; Bachhav et al., 2022; Santiago-Lopez et al., 2022a; Carrillo et al., 2022; Kroukamp et al., 2022; Navarro-Tapia and Perez-Torrado, 2022). Although some effective compounds from chemical libraries or native products have been screened out, the direct targets and action mechanisms of these compounds are unclear. This may be one of the reasons hindering the progress of clinical research, and more efforts are still required to resolve this problem.

On the other hand, ER stress has been shown to be a regulatory target to rescue neuronal death in different experimental models. Neurotoxin-based rodents and cell lines are the most widely used models for drug screening and investigation; however, they still cannot completely reflect the pathophysiological state of human DA neurons in PD. The use of PD patient iPSCs to differentiate midbrain DA neurons may reverse this dilemma. Since these iPSCs come from patients’ somatic cells, they have the same genetic background as humans (Kiskinis and Eggan, 2010; Hartfield et al., 2012). However, the application of iPSCs still has some limitations. For instance, iPSCs undergoing dedifferentiation lose phenotypic and molecular features associated with aging, which renders this model unable to mimic aging-related disease (Puri and Wagner, 2023). Moreover, the risk and severity of PD are not only related to age but also related to gender (Elbaz et al., 2000; Accolla et al., 2007). The gender difference may pose some difficulties when using experimental PD animal models to explore drug effects and mechanisms (Zarate et al., 2021). Although some researchers have made an iPSC model that can simulate gender differences, the actual application effect of this model still lacks supporting data (Waldhorn et al., 2022). The applicability and accuracy of the experimental model are an important basis for the study of pathogenesis mechanisms, drug screening, and clinical trials. More efforts are still needed to improve the iPSC-derived neuron model or find a better alternative model.

## 6 Conclusion and perspectives

ER stress is one of the important pathological phenomena of PD, and it interacts with various other pathological events such as mitochondrial dysfunction, autophagy, neuroinflammation, and oxidative stress to jointly promote the occurrence and development of PD. However, the precise mechanism is unclear. Thus, the association of ER stress with other PD phenotypes is worth exploring. In addition, non-motor symptoms including emotion, cognition, sleep, hyposmia, and vision in PD have attracted more and more attention (Fuchigami et al., 2023; Sanchez-Saez et al., 2023). Exploring the role of ER stress in these non-motor symptoms can provide a comprehensive understanding of PD. Meanwhile, a large number of studies have shown that targeting ER stress can effectively protect midbrain DA neurons from apoptosis and relieve PD symptoms in different PD models. In addition, reliable drug screening models targeting ER stress are increasingly available. Future research directions can focus on the confirmation of clear and direct targets of these candidate drugs, the precise control of UPR regulation, and the improvement of experimental models.
